# Impact of financial compensation on enrollment and participation in a remote, mobile-app based research study

**DOI:** 10.1017/cts.2024.515

**Published:** 2024-04-05

**Authors:** Shelby Meier, Alex Cheng, Maeve Tischbein, Cathy Shyr, Rebecca N. Jerome, Terri L. Edwards, Mary Stroud, Consuelo H. Wilkins, Paul A. Harris

**Affiliations:** 1 Vanderbilt Institute for Clinical and Translational Research, Vanderbilt University Medical Center, Nashville, TN, USA; 2 Department of Biomedical Informatics, Vanderbilt University Medical Center, Nashville, TN, USA; 3 Office of Health Equity, Vanderbilt University Medical Center, Nashville, TN, USA

**Keywords:** Clinical trials, financial incentive, motivation, participant recruitment, participant compensation

## Abstract

**Background:**

There is no consensus on how to determine appropriate financial compensation for research recruitment. Selecting incentive amounts that are reasonable and respectful, without undue inducement, remains challenging. Previously, we demonstrated that incentive amount significantly impacts participants’ willingness to complete various hypothetical research activities. Here we further explore this relationship in a mock decentralized study.

**Methods:**

Adult ResearchMatch volunteers were invited to join a prospective study where interested individuals were given an opportunity to view details for a study along with participation requirements, then offered a randomly generated compensation amount between $0 and $50 to enroll and participate. Individuals agreeing to participate were then asked to complete tasks using a remote mobile application (MyCap), for two weeks. Tasks included a weekly survey, a daily gratitude journal and daily phone tapping task.

**Results:**

Willingness to participate was 85% across all incentive levels but not significantly impacted by amount. Task completion appeared to increase as a function of compensation until a plateau at $25. While participants described the study as low burden and reported that compensation was moderately important to their decision to join, only 31% completed all study tasks.

**Conclusion:**

While offering compensation in this study did not have a strong effect on enrollment rate, this work provides insight into participant motivation when joining and participating in studies employing mobile applications.

## Introduction

The collective success of healthcare research efforts in the United States (U.S.) relies on the ability of research teams to recruit and retain participants in studies. Numerous recruitment strategies are described in the literature [1–5]. The use of financial incentives, as an approach for compensating participant time and effort as well as a show of respect for their contribution to the healthcare research, is one mechanism that receives considerable interest in terms of improving recruitment [6–8]. While some researchers have explored interaction between demographic factors such as income or race and ethnicity on compensation preferences [9–11], there remains a need for further exploration of this issue, including variability in response and preferences among different demographic groups. There is also no clear consensus regarding the best approach for determining financial compensation. This lack of guidance and nascent evidence base present a challenge to researchers seeking to determine respectful incentives that improve study enrollment, engagement, and retention, but do not provide undue inducement [9–11].

The Recruitment Innovation Center (RIC), funded by the National Center for Advancing Translational Sciences (NCATS), develops evidence-based recruitment and retention solutions to improve the quality of clinical trials. Previously, we evaluated the relationship between financial incentive amount and hypothetical willingness to participate in various research scenarios [12]. We determined that willingness to participate was positively correlated with compensation amount and that higher-burden tasks generally required higher compensation amounts. While our previous study effectively queried participants about their willingness to participate in a variety of research tasks, it was limited in that all scenarios were hypothetical. Additionally, participant follow-through and actual performance of presented study tasks were not assessed.

The current work expands on our previous efforts by both assessing the relationship between compensation amount and willingness to join a research study, as in our original work, and the added dimension of participant adherence to study tasks within a decentralized mock study.

## Methods

### Study population

Participants were recruited from ResearchMatch, a national, nonprofit, volunteer-to-researcher matching platform that includes more than 150,000 volunteers [13]. Individuals aged 18+ years with no reported health conditions (i.e., “healthy individuals”) were invited to join. The racial and ethnic enrollment goal for this study was based on the makeup of the U.S. (59% White, 14% Black or African American or African, 19% Hispanic or Latino, 6% Asian, and 2% American Indian or Alaska Native) [14]. To ensure a racially and ethnically diverse sample, the demographic makeup of respondents was reviewed after each wave of study invitations was sent. Imbalances in enrollment of underrepresented groups in this study were iteratively targeted in subsequent invitation waves (Appendix Table 1).

### Study design

**Figure 1.**
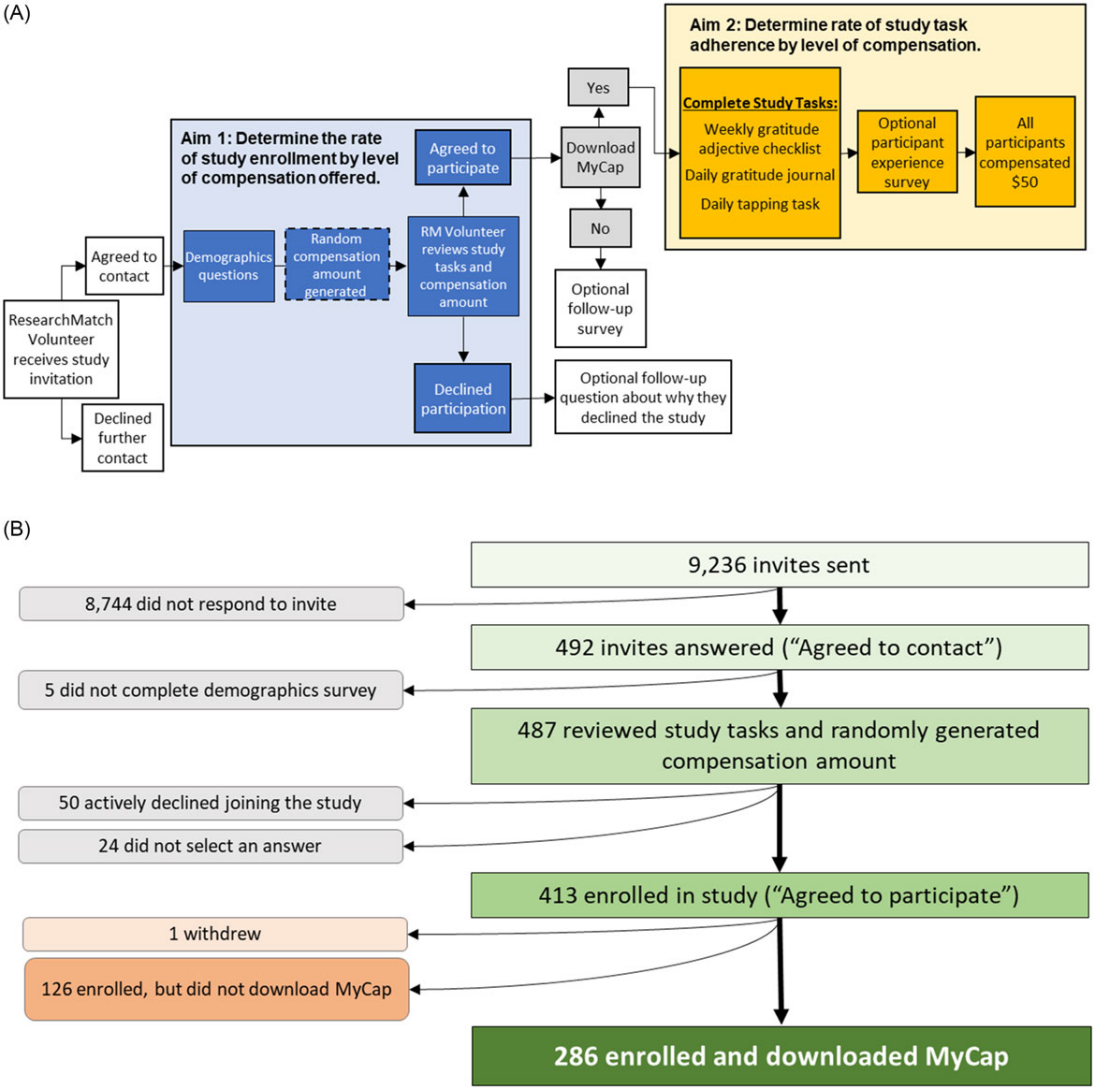
Participant flow for the study. (A) Schematic showing participant experience from invitation to study completion. The random compensation offer was generated after participant demographics were collected (dotted box outline) and was shown to volunteers at the same time as the study description. (B) Enrollment flow from invitation to enrollment and downloading the study app, MyCap.

This study was approved by the Vanderbilt Institutional Review Board as exempt research (IRB #221043). Participants used MyCap [15], a participant-facing data collection mobile application that securely transmits data to and from REDCap [16,17], to perform study tasks. We implemented study tasks of varying frequency and type for participants to complete: a weekly Gratitude Adjective Checklist [18], a daily gratitude journal, and a daily tapping task [15]. Figure 1 details the participant flow for this study. Volunteers who responded positively to the study invitation were immediately redirected to our REDCap-based survey for enrollment, which included a brief demographic questionnaire that queried age group, gender identity, race and ethnicity, educational level, employment status, and annual household income. All respondents who completed this questionnaire were included in the denominator for participant enrollment rate.

Respondents were then provided an e-Consent form describing the study and a randomly generated promised compensation amount between $0 and $50, with each intermediate price point increasing by increments of $5. Respondents were informed that they would need to complete all tasks in order to receive compensation. After reviewing the e-Consent form and compensation amount, respondents were asked whether or not they would join the study. The randomly generated amount communicated to participants was an IRB-approved deception, as all participants agreeing to participate in the study and download MyCap were compensated equally at the end of the study ($50). As it was important to know if the amount of money offered impacted decisions to join and adhere to the study, volunteers consented to join a study with a stated purpose of understanding “how paying people for being in a study affects their willingness to join and their participation throughout the study” and told that the study contained element of deception, which would be revealed upon completion. After the study, participants received an email explaining the nature of the deception.

For those agreeing to join, a custom REDCap external module was used to randomize participants to compensation amounts stratified by gender (woman, man, and non-woman and non-man identities), race/ethnicity (Black, White, and non-Black and non-White racial and ethnic identities) and income (<$65,000/year, ≥ $65,000/year, no answer). These groupings were determined by our study team to ensure randomization was relatively balanced across these demographic categories.

Upon indicating they would like to join and participate in the decentralized data collection study, participants were asked to download MyCap and complete study tasks in this app over a 14-day period. Participants were then asked to complete an optional survey on their experience one week after the 14-day task period. This follow-up survey explored perceived study burden, the impact of compensation on their decision to join the study, whether the amount of compensation offered was believed to be fair, and, if not, what amount they thought to be fair. Qualitative questions about the MyCap app were also asked.

To gain a better understanding of the reasons people opted NOT to join the study, we asked volunteers who declined the study to share their reasoning. Respondents who did not join the data collection study were offered the opportunity to enter a drawing for a $50 gift card.

### Data analysis

We sought to assess the impact of offered compensation on participant willingness to join (herein, referred to as enrollment for the purposes of this mock study), with a logistic regression with enrollment in the study as the outcome. While other logistic regression studies follow a “10 to 1” rule, where 10 samples are needed for every independent variable in the regression, we were more conservative and aimed to recruit 15 participants per degree of freedom [19]. With 11 price points ($0 - $50, $5 increments), three race categories, four age categories, and income as an ordinal variable, we sought at least 300 ResearchMatch respondents (i.e., volunteers that provided demographic information, read the study description and randomly generated compensation offer, and expressed if they wanted to participate in the study; Aim 1 in Fig. 1). The number of respondents was not limited to 300 volunteers; study invitations were sent in waves and enrollment concluded following the wave of invitations in which 300 participants were obtained. The primary null hypothesis of this aim was that there is no statistically significant association between offered financial incentive and willingness to participate in the study.

For assessing the impact of compensation offered on dataset completeness (i.e., participants downloading MyCap and taking part in study tasks; Aim 2 in Fig. 1A), the contents of participant responses for each study task were not analyzed; rather we looked at the presence or absence of a response. A 2-sample test for equality of proportions with continuity correction was used to assess the proportion of participants that said yes to the study invitation and downloaded MyCap for each race/ethnicity category as compared to white participants.

Loess curves were used to visualize the effect of incentive amount on study participation rate and dataset completeness. We ran a logistic regression to determine whether any factors contributed significantly to the participation rate. We used a one-sided, two-sample test for equality of proportions with continuity correction to compare the proportion of tasks completed between two compensation amounts. To assess the effect of study compensation across task types (daily vs. weekly study tasks), we used a logistic mixed effects model with a random intercept. Specifically, we regressed task completion (yes/no) on compensation amount, task type (daily/weekly frequency) and an interaction term between compensation amount and task type. To analyze retention among participants who agreed to join the study, we plotted Kaplan-Meier curves for the last day that each participant completed any study task by compensation amount [20,21]. All statistical analyses were run using R version 4.3.0 with data pulled directly through the REDCap Application Programming Interface.

## Results

We sent a total of 9,236 invitations to ResearchMatch volunteers and received 492 expressions of interest. Of those interested, 413 were enrolled (i.e., said “yes” to joining) in the study (Fig. 1). One participant withdrew after enrollment; no reason for withdrawal was given. Of the 412 remaining enrolled participants, 286 downloaded the required MyCap study app. We noted an increased proportionality of Black (65%, 95% CI 60%–88%) and Asian (76%, 95% CI 55%–74%) respondents that downloaded MyCap relative to White participants (55%, 95% CI 49%–61%); however, only the latter group reached statistical significance (*p* = 0.03). Table 1 summarizes participant demographic data.

**Table 1.** Demographics of participants. Self-reported characteristics of ResearchMatch volunteers that responded to the study invitation, participated in or declined participation.

	Responded to invitation	Said yes + downloaded MyCap	Declined participation
**Characteristic**	*n* = 492	*n* = 286	*n* = 50
**Age**	***N* (%)**	***N* (%)**	***N* (%)**
18–29	118 (23.9)	71 (25)	6 (12)
30–49	186 (37.8)	127 (44.7)	13 (26)
50–64	104 (21.1)	52 (18.3)	13 (26)
65–74	56 (11.3)	25 (8.8)	10 (20)
75 and older	21 (4.2)	8 (2.8)	8 (16)
Prefer not to answer	1 (<1)	1 (<1)	0 (0)
Missing	6 (1.2)	0 (0)	0 (0)
**Gender Identity**			
Woman	267 (54.3)	151 (53.2)	32 (64)
Man	211 (42.9)	127 (44.7)	18 (36)
Nonbinary	7 (1.4)	5 (1.8)	0 (0)
Transgender	0 (0)	0 (0)	0 (0)
Did not identify with any options listed	1 (<1)	1 (<1)	0 (0)
Prefer not to answer	0 (0)	0 (0)	0 (0)
Blank	6 (1.2)	0 (0)	0 (0)
Race/Ethnicity*			
American Indian/Alaska Native	11	6	0
Asian or Asian American	34	26	1
Black, African American, African	103	67	5
Hispanic, Latino, Spanish	114	63	9
Middle Eastern, North Africa	3	3	0
Native Hawaiian, other Pacific Islander	0	0	0
White, Caucasian	278	153	39
Prefer not to answer	5	1	1
**Highest level of education**			
Never attended school or only attended kindergarten	0 (0)	0 (0)	0 (0)
Grades 1–4 (Elementary School)	1 (<1)	1 (<1)	0 (0)
Grades 5–8 (Middle School)	0 (0)	0 (0)	0 (0)
Grades 9–11 (Some high school)	1 (<1)	1 (<1)	0 (0)
Grade 12 or GED (High school graduate)	25 (5.1)	16 (5.6)	1 (2)
College 1–3 years (Some college, Associate’s degree or technical school)	109 (22.2)	56 (19.7)	11 (22)
College 4 years or more (College graduate)	187 (38)	109 (38.4)	18 (36)
Advanced degree (Master’s, Doctorate, etc.)	161 (32.7)	101 (35.6)	19 (38)
Prefer not to answer	2 (<1)	0 (0)	1 (2)
Missing	6 (1.2)	0 (0)	0 (0)
**Employment status**			
Employed full time (32+ hours a week)	248 (50.4)	162 (57)	20 (40)
Employed part time (less than 32 hours per week)	72 (14.6)	36 (12.7)	7 (14)
Unemployed	41 (8.3)	26 (9.2)	3 (6)
Retired	67 (13.6)	27 (9.5)	17 (34)
Unable to work due to disability	32 (6.5)	19 (6.7)	3 (6)
Other	26 (5.3)	14 (4.9)	0 (0)
Prefer not to answer	0 (0)	0 (0)	0 (0)
Missing	6 (1.2)	0 (0)	0 (0)
**Household income**			
Less than $35,000	93 (18.9)	56 (19.7)	6 (12)
$35,000–$64,999	110 (22.4)	59 (20.8)	9 (18)
$65,000–$99,000	115 (23.4)	78 (27.5)	12 (24)
$100,000 or more	139 (28.3)	75 (26.4)	16 (32)
Prefer not to answer	29 (5.9)	16 (5.6)	7 (14)
Missing	6 (1.2)	0 (0)	0 (0)

* Numbers do not tally as respondents were able to select all categories that they felt applied to them.

**Figure 2.**
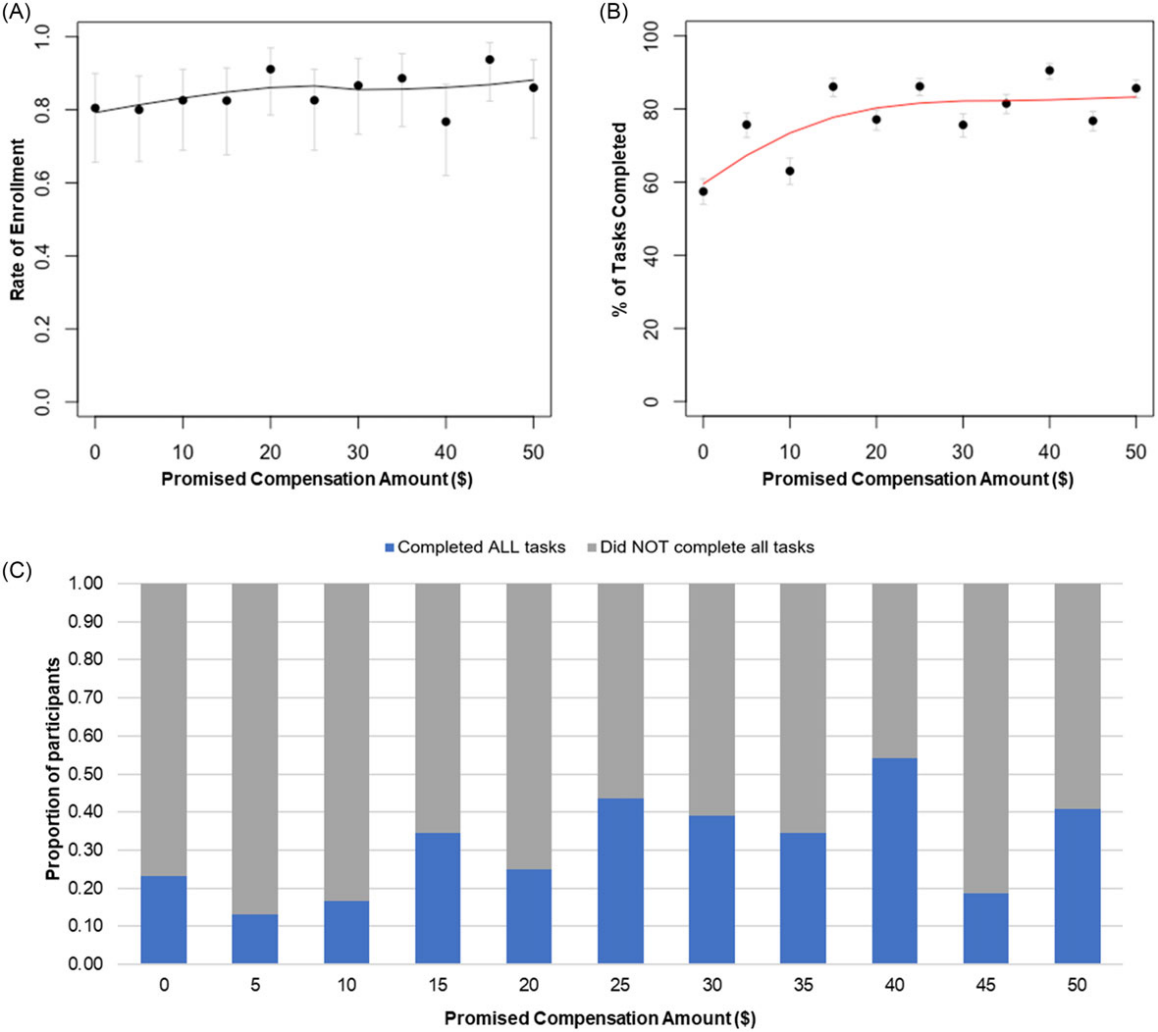
Participant enrollment and adherence to mock study tasks. (A) Rate of participant enrollment in the study with the line representing the Loess curve for rate of enrollment by promised compensation amount for the whole study group. Dots are mean values at each promised compensation amount, and bars are 95% Wilson confidence intervals. *n* = 486 (as enrollment rate was calculated from those who said yes OR no). (B) Mean task completion; red line representing the Loess curve of total task completion by compensation amount, *n* = 286. Bars depicting the 95% Wilson confidence interval. (C) Proportion of participants that did or did not complete ALL study tasks for each promised compensation amount, *n* = 286.

For all price points, the enrollment rate remained high (∼80%–90%; Fig. 2A) with a high degree of overlap in the 95% confidence intervals, and there was insufficient evidence to substantiate a statistically significant relationship between enrollment and compensation (*p* > 0.05 based on a Wald test). No factors, including age, race, income, and promised compensation amount, were statistically significant contributors to the participant’s decision to join our study in the logistic regression.

Between $0 and $25, task completion increased from 60 to 80%, and this difference was statistically significant (*p* < 2e–16 based on a two-sample proportion test). For promised compensation offers greater than $25, task completion plateaued around 80% (Fig. 2B). When separated by weekly (gratitude adjective checklist) or daily tasks (tapping tasks and gratitude journal entries), the effect of compensation was not statistically different (*p* = 0.09). Overall, 31% of participants who agreed to participate completed all study tasks. Only one incentive amount ($40) had more than 50% of participants complete all tasks (54%; Fig. 2C). When evaluating participant retention (defined as the last day of recorded study activity) using Kaplan-Meier curves we observed that retention was not equal between compensation groups (*p* = 0.0019).

### Post-study participant perspectives

**Figure 3.**
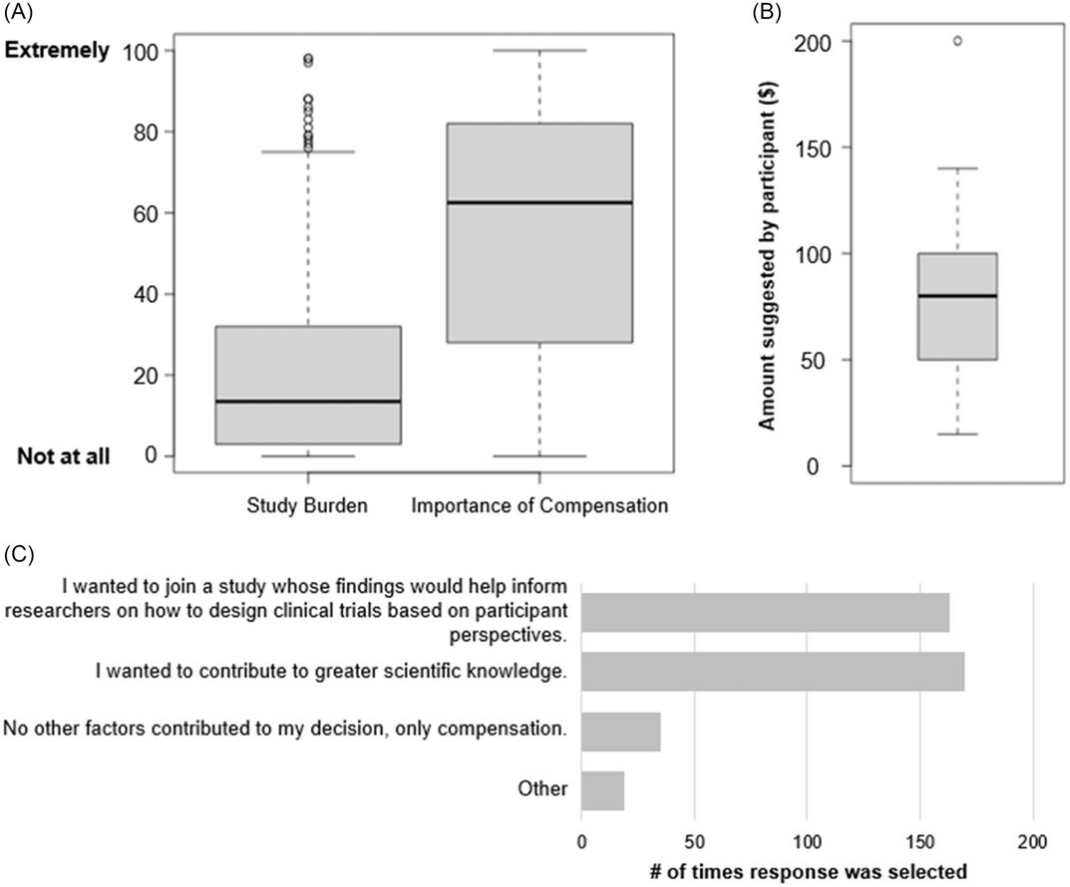
Participant experience survey responses. (A) Box and whisker plot for responses around perceived study burden and importance of compensation when joining this study, *n* = 265. (B) Box and whisker plot of compensation amounts suggested by participants that believed their initial compensation offer was unfair, *n* = 13 (3 respondents did not provide a suggested compensation amount). Dots are individual response values. (C) Additional reasons participants chose to be in this study (participants could select more than one answer when responding), *n* = 265.

After the 14-day study period, all participants were invited to complete a brief questionnaire about their study experience. We focused on participant perceptions around burden, the importance of compensation, and additional motivating factors related to enrollment in this study. Of the 286 participants who enrolled and downloaded MyCap, 265 responded to this optional questionnaire. The majority of participants (*n* = 193, 73%) found the study to be low burden (rated ≤ 30 on a slider scale from 0 to 100) (Fig. 3A). Even with this subjectively rated “low burden” study, compensation was of moderate importance in the decision-making process (rated between 31 and 69 on a slider scale from 0 to 100) (Fig. 3A). While most study participants believed that their compensation offer was fair, a small number of participants (*n* = 16, data not shown) disagreed. All participants who believed their offer was unfair were asked to suggest a fair compensation amount for the study. The amounts suggested ranged from $15–$200, with the average being $80.96 (Fig. 3B) and one participant saying any amount other than $0 was fair. Participants were also asked to share any motivating factors that did not involve compensation. Desire to contribute to greater scientific knowledge and help researchers understand the importance of compensation in clinical trials were most frequently selected by participants (Fig. 3C). 35 of the 265 respondents indicated that compensation was the only factor contributing to their decision.

### Respondents who did not complete all steps to join the study or actively declined participation

Of the 492 expressions of interest in our study from the initial ResearchMatch invitees, 126 said yes to joining the study but did not download the required MyCap study app and 50 actively declined participation by selecting “no” when asked if they would join the study (Fig. 1B). Table 1 summarizes the demographics of those who actively declined participation.

We sent a brief survey to the 126 participants who did not download MyCap and received 25 responses (20%). This follow-up survey focused on perceived obstacles around downloading the app. Having to download the app itself was the main reason that 54% of the respondents reported they did not continue with the study. For participants who said downloading the app was NOT the main reason for their discontinuation in the study, additional reported obstacles such as forgetting about the study and technical difficulties were reported (data not shown).

**Figure 4.**
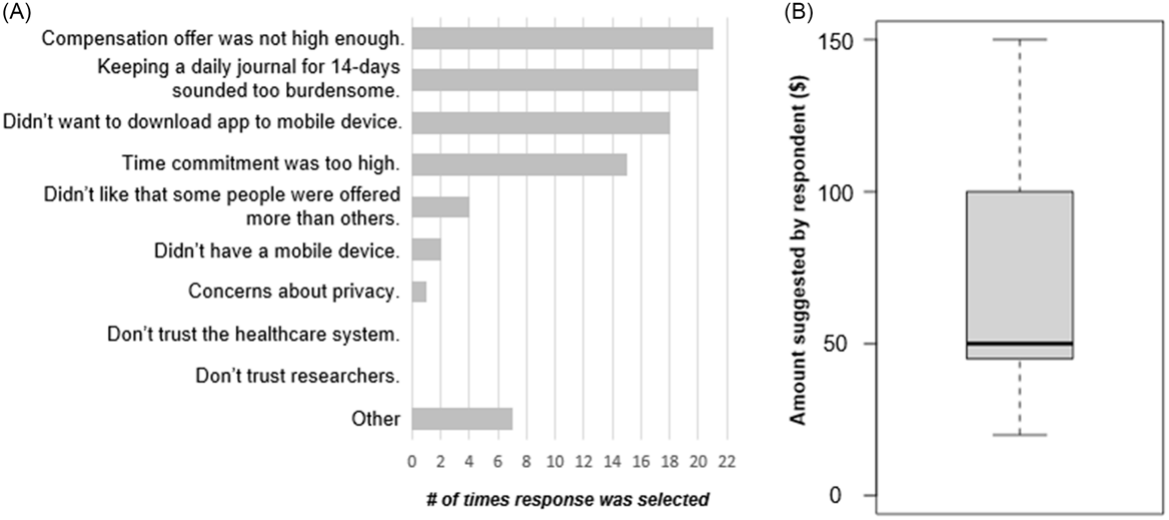
Reasons for study declination by participants. (A) Responses to the question “Would you please share any reasons why you didn't want to join the study?” from respondents that turned down the study, *n* = 50. Respondents could select all options that applied to them. (B) Compensation amount suggestions from respondents that believed their compensation offer was not high enough, *n* = 19.

For the 50 respondents who actively declined participation, we asked them to share their reasoning; all those who declined participation completed this optional follow-up. The most frequently selected reasons for actively declining participation were related to compensation amount, participant burden, and not wanting to download an app (Fig. 4A). We further investigated the compensation amount offered to respondents that had indicated “Compensation offer wasn't high enough.” The amount offered at enrollment was varied, but the majority received offers of $15 or less. The proportion of those who received offers of $15 or less and said no (15/50; 30%) was similar to the proportion of those who received offers of $15 or less and said yes (94/286; 33%). We also asked these respondents what compensation amount would have been acceptable, and found the mean suggested amount to be $60 (Fig. 4B). Respondents that selected “Other” were asked to clarify their reason in a free response text box. A common theme within those explanations was a dislike of the study containing elements of deception (the study description shown to participants in the e-Consent document included language letting them know there were elements of deception in the study, but those elements did not influence study activities or risk of the study and the elements of deception would be revealed after completing the study).

## Discussion

### Summary of study findings

In this study, we built upon previous work [12] by exploring the potential correlation between promised compensation amount and participant willingness to join a research study as well as adherence to a study task schedule. We found that level of compensation did not have a significant effect on enrollment as expected over the range tested. Future iterations of this study may increase the range of compensation or decrease the workload of the study to determine if there is more differentiation in enrollment rates by compensation amount. We also observed that as the promised compensation amount increased, the number of overall tasks completed appeared to likewise increase until participants were offered $25 or more (Figs. 2b,c). Though participants subjectively reported the mock study activities to be low burden, no promised compensation amount resulted in more than 54% of participants completing all study tasks, which is relatively concordant with the 44%–46% completion rates reported for other online studies [22,23]. For researchers seeking to determe the “right” level of compensation or an estimation of participant engagement for a given compensation amount, this evidence-based approach using research participants as key informants may be a useful strategy.

### Comparison of our findings to previous work study

In comparison to our previous work, where participation rates with increasing amounts of compensation nearly all hypothetically-proposed study tasks [12], our key finding here differs: there was no significant effect of compensation amount on participant willingness to join. Both studies were recruited from ResearchMatch, but there were notable differences between the investigations. The original study focused entirely on hypothetical scenarios; participants were never asked to actually complete any tasks, but rather only *consider* completing a single task (i.e., Would you keep a daily record of how much water you drink for one week and discuss it with clinic staff for $X?). Focusing on a single task, rather than multiple activities within a study, could allow participants to consider their decision more clearly without having to weigh multiple options. Also, the tasks presented in the original study were a mix of remote and “in-person” study activities. For some participants, having to travel for a study visit could be a major burden and the amount of compensation promised may have figured more prominently in their decision. The current study was reported as generally low burden by participants and the compensation offered may have had less of an effect, potentially as there were few perceived obstacles in joining. For both studies we recognize the hypothetical or mock research tasks examined may not directly relate to a given participant’s health or healthcare and that participation or completion of study tasks may differ when volunteers are asked to report data that is more sensitive or of greater personal relevance. We may expand use of this platform to additional research applications and scenarios in the future to further add to our understanding of participant compensation expectations across diverse study requirements.

Research studies commonly compensate participants for various research-related tasks, but the appropriateness of compensation amounts remains a topic of debate. A meta-analysis of the effect of compensation on enrollment in randomized clinical trials showed that offering compensation significantly increased the rate of response and consent [7]. Additionally, other investigators have reported compensation as a motivating factor for participants, but not in a way that suggested undue influence or inducement [24]. The RETAIN study [25], led by investigators at the University of Pennsylvania, found that compensation significantly increased enrollment rates for a smoking cessation trial, but not for an ambulation intervention trial. In both trials, there was no evidence that compensation offers produced undue influence even with offers up to $500 (smoking cessation trial) and $300 (ambulation trial). They concluded that studies offering compensation are not unethical, but that the effects of incentives on enrollment may not be consistent across all clinical trials [9]. In contrast to these findings, our data showed only a slight, nonsignificant, positive slope between promised compensation and enrollment rate. However, participants’ responses in the post-study experience survey showed that, subjectively, compensation was of moderate importance to participants. This demonstrates that, at least in this study design with this population, the amount of compensation may have mattered but did not have a major impact on a participant’s willingness to enroll. This finding is in line with the conclusions from the RETAIN study: the effects of compensation may vary between trials. Taken together, these data suggest the specific amount offered to a participant doesn't need to be exactly “perfect,” but that the act of offering some level of compensation is, itself, critical. This is supported in the literature, especially in studies where participants are expected to face co-payments or other obstacles to participation [7,26,27], and as an approach for demonstrating respect for and value of the participants in the study [28]. While our evidence-based findings can help inform compensation decisions in clinical trial design, we recommend through our additional efforts through the RIC that study teams use Participant/Patient Advisory Groups to directly ask participants about adequate compensation for their specific study whenever possible.

### Potential limitations

The study population was sourced from ResearchMatch and we recognize there is likely some degree of self-selection among the registry volunteers that were willing to participate in this study. Consistent with the ResearchMatch population, the study cohort is highly educated (>82% have at least some college-level or more education) and is employed full or part time (>60%). However, participant responses to our invitations skewed younger (majority <49 years of age), a trend that was enhanced further among those participants who proceeded to download MyCap. Further, ResearchMatch volunteers have already self-selected for interest in research by their initial joining of this community, thus are likely to have a history of research participation and associated positive attitudes. Overall, these characteristics may somewhat limit the generalizability of our results to a more heterogeneous population. Moreover, this study was only available in English. We acknowledge the need for a multimodal and multilingual approach to participant recruitment in order to mitigate selection bias inherent to any single strategy. Future efforts will seek to include populations more representative of the general public (e.g., CINT database [29]) as well as populations outside of research registries (which may more accurately reflect the attitudes of the general American population).

For this study, participants were told they would receive a random amount between $0-50 for participating when, actually, all who enrolled and downloaded MyCap received a $50 gift card. This “deception” method was to ensure ethical responsibility by compensating all participants equally for the same amount of participation. From a budgeting standpoint, the need to compensate every participant with the highest amount prevented us from testing a wider range of values (for example $0-$100) where we might have been able to detect a difference in enrollment rate. Deception was not a major reason endorsed by those declining the study, possibly due to the research-minded disposition of the ResearchMatch population and the low-risk nature of the study. Such research-mindedness may have also contributed to the lack of differences in enrollment based on compensation in this study. It is possible that the deception may have also had the opposite effect, artificially elevating the enrollment rate for lower dollar amounts as volunteers considering this study about study compensation may have suspected that they would get the full $50 regardless of what was offered in the consent form. However, we acknowledge that deception could be triggering for people from marginalized backgrounds that have been historically exploited, including undisclosed and harmful deception in past research [30,31]. Participant concerns around deception remain a general and important consideration in the design of future and/or replicate studies, especially among populations where privacy and/or trust are of known concern.

By utilizing the MyCap study app, we were able to conduct this study in a fully remote environment. Since the onset of the COVID-19 pandemic, the literature indicates a growing number of studies incorporating remote aspects [32–35]. While there is evidence that remote data collection reduces the burden for participants [36] and makes studies more accessible [37], this does not mean there aren’t obstacles or challenges for study teams to consider when designing a remote trial. As demonstrated by this study, one of the top-reported reasons for study declination was “didn’t want to download app to mobile device’ and, of those that responded to the study invite but did not download MyCap, having to download the app was the main reason for not continuing with the study. Though mediation of app-related study declination was not examined here, the consideration of a participant’s willingness to download a study app and overcome technical difficulties as well as the provision of clear instructions (i.e., infographic, step-by-step instruction handout with images, or a short video) are in line with the findings and experience of the RIC.

This study did not investigate the effects of prorating payments or other engagement/retention strategies (such as reminders or gamification) that could impact a participant’s decision to enroll. Prorating payments (i.e., paying participants in small increments as they complete tasks, rather than one lump sum at the end of the study) is recommended to encourage participants to complete checkpoints, especially in longer studies, to mitigate any participant-incurred burdens related to costs incurred by their decision to remain in the study [38,39]. Our study was relatively short and rated by participants to be low burden, so it is possible that prorating payments would not have had any effect. Additionally, our study had a fairly low rate of study declination (∼10%) and participant-provided reasons for turning down the study indicated that it was due to the amount rather than the timing of the payment. Early engagement strategies, such as building trust, improving participant comprehension of the study, and appropriately framing risks and benefits have been shown to have a significantly positive effect on recruitment in some studies [40]. Our individual study relied heavily on previous work done by the ResearchMatch group to establish trust with our participants. While not a part of this study, we drew upon the experience of RIC to build trust by making the study easy to understand and engaging our Community Advisory Board as to the presentation and readability of the e-Consent document used. Additional studies could further explore how to best communicate elements of deception within a study without eroding any trust that is already built.

## Conclusions

Together, this study supports compensation as an important factor considered by participants when choosing to enroll, but that the amount itself may be less important than anything.

## Supporting information

Meier et al. supplementary material Meier et al. supplementary material

## Supplementary material

The supplementary material for this article can be found at https://doi.org/10.1017/cts.2024.515.
