# Supplementary material for: Impact of financial compensation on enrollment and participation in a remote, mobile-app based research study
Source: J Clin Transl Sci. 2024 Apr 5;8(1):e75. doi: 10.1017/cts.2024.515 (PMC11075110; doi:10.1017/cts.2024.515)
Supplement: Meier et al. supplementary material [file S2059866124005156sup001.docx]

# **Appendix** for *Impact of financial compensation on enrollment and participation in a remote, mobile-app based research study*

**Table 1. Demographic breakdown of ResearchMatch volunteers sent and responding to a study invitation by invitation wave.** The characteristics of the invited cohort (i.e., sex, race and ethnicity) reflect the variables available for selection by researchers using ResearchMatch at the time the study occurred. Waves of invites were generated based on the desired demographic characteristics to ensure the inclusion of underrepresented groups; dashes (-) indicate variables that were not included when selecting a demographic-driven subset for a given wave. Approximately 4-8 days after each round of invitations (the time frame for highest general response rate in ResearchMatch), self-reported demographics were used to inform the selection of the next invitation wave. Note: the characteristics of respondents when viewed by wave represent approximate results as there was a small amount of bleed-through occurring when a volunteer from a previous wave responded to an invite after the 4-8 day period from which it was initially sent.

| Characteristics of Invited Cohort | Wave 1 (252 Total) | Wave 2  (2246 Total) | Wave 3  (2250 Total) | Wave 4  (1500 Total) | Wave 5  (2988 Total) |
| --- | --- | --- | --- | --- | --- |
| Sex Assigned at Birth |  |  |  |  |  |
| Male | 125 | 2246 | 0 | 750 | 1488 |
| Female | 125 | - | 2250 | 750 | 1500 |
| Intersex | 2 | - | - | - |  |
| Race |  | 1496 | 1500 |  |  |
| Black, African American, or African | 90 |  |  | - | - |
| Asian | 52 |  |  | - | - |
| American Indian or Alaska Native | 8 |  |  |  | - |
| Native Hawaiian or other Pacific Islander |  |  |  | - | - |
| Multi-Racial | - |  |  | - | - |
| Other | - |  |  | - | - |
| White | 102 | - | - | - | 2988 |
| Ethnicity |  |  |  |  |  |
| Hispanic or Latino | - | 750 | 750 | 1500 | - |
| Not Hispanic or Latino | - | - | - | - | - |
| Characteristics of Respondents | **Wave 1**  **(9 Total)** | **Wave 2**  **(96 Total)** | **Wave 3**  **(103 Total)** | **Wave 4**  **(65 Total)** | **Wave 5**  **(219 Total)** |
| Gender Identity |  |  |  |  |  |
| Woman | 4 | 6 | 94 | 33 | 130 |
| Man | 5 | 86 | 4 | 31 | 85 |
| Nonbinary | 0 | 2 | 2 | 0 | 3 |
| Transgender | 0 | 0 | 0 | 0 | 0 |
| Did not identify with any options listed | 0 | 0 | 1 | 0 | 0 |
| Prefer not to answer | 0 | 0 | 0 | 0 | 0 |
| Blank | 0 | 2 | 2 | 1 | 1 |
| Race/Ethnicity** |  |  |  |  |  |
| American Indian/Alaska Native | 0 | 5 | 3 | 1 | 2 |
| Asian or Asian American | 0 | 8 | 23 | 1 | 2 |
| Black, African American, African | 5 | 44 | 39 | 5 | 10 |
| Hispanic, Latino, Spanish | 0 | 24 | 36 | 46 | 8 |
| Middle Eastern, North Africa | 0 | 0 | 1 | 0 | 2 |
| Native Hawaiian, other Pacific Islander | 0 | 0 | 0 | 0 | 0 |
| White, Caucasian | 4 | 28 | 10 | 35 | 201 |
| Prefer not to answer | 0 | 1 | 0 | 1 | 3 |

*Note: Numbers do not tally to total number of respondents as volunteers were able to select all categories that they felt applied to them.
